# ERICA: smoking prevalence in Brazilian adolescents

**DOI:** 10.1590/S01518-8787.2016050006741

**Published:** 2016-02-02

**Authors:** Valeska Carvalho Figueiredo, André Salem Szklo, Letícia Casado Costa, Maria Cristina C Kuschnir, Thiago Luiz Nogueira da Silva, Katia Vergetti Bloch, Moyses Szklo

**Affiliations:** I Centro de Estudos sobre Tabaco e Saúde. Escola Nacional de Saúde Pública Sergio Arouca. Fundação Oswaldo Cruz. Rio de Janeiro, RJ, Brasil; II Divisão de Epidemiologia. Coordenação Geral de Ensino e Pesquisa. Instituto Nacional de Câncer José Alencar Gomes da Silva. Rio de Janeiro, RJ, Brasil; III Serviço de Edição e Informação Técnico Científica. Coordenação de Prevenção e Vigilância. Instituto Nacional de Câncer José Alencar Gomes da Silva. Rio de Janeiro, RJ, Brasil; IV Faculdade de Ciência Médicas. Núcleo de Estudos da Saúde do Adolescente. Universidade do Estado do Rio de Janeiro. Rio de Janeiro, RJ, Brasil; V Instituto de Estudos em Saúde Coletiva. Universidade Federal do Rio de Janeiro. Rio de Janeiro, RJ, Brasil

**Keywords:** Adolescent, Tobacco use, epidemiology, Prevalence, Survey

## Abstract

**OBJECTIVE:**

To estimate the prevalences of tobacco use, tobacco experimentation, and frequent smoking among Brazilian adolescents.

**METHODS:**

We evaluated participants of the cross-sectional, nation-wide, school-based Study of Cardiovascular Risks in Adolescents (ERICA), which included 12- to 17-year-old adolescents from municipalities of over 100 thousand inhabitants. The study sample had a clustered, stratified design and was representative of the whole country, its geographical regions, and all 27 state capitals. The information was obtained with self-administered questionnaires. Tobacco experimentation was defined as having tried cigarettes at least once in life. Adolescents who had smoked on at least one day over the previous 30 days were considered current cigarette smokers. Having smoked cigarettes for at least seven consecutive days was an indicator for regular consumption of tobacco. Considering the complex sampling design, prevalences and 95% confidence intervals were estimated according to sociodemographic and socio-environmental characteristics.

**RESULTS:**

We evaluated 74,589 adolescents. Among these, 18.5% (95%CI 17.7-19.4) had smoked at least once in life, 5.7% (95%CI 5.3-6.2) smoked at the time of the research, and 2.5% (95%CI 2.2-2.8) smoked often. Adolescents aged 15 to 17 years had higher prevalences for all indicators than those aged 12 to 14 years. The prevalences did not differ significantly between sexes. The highest prevalences were found in the South region and the lowest ones, in the Northeast region. Regardless of sex, the prevalences were found to be higher for adolescents who had had paid jobs, who lived with only one parent, and who reported having been in contact with smokers either inside or outside their homes. Female public school adolescents were found to smoke more than the ones from private schools.

**CONCLUSIONS:**

Tobacco use among adolescents is still a challenge. Intending to reduce the prevalence of tobacco use among young people, especially the ones under socioeconomic vulnerability conditions, Brazil must consolidate and increase effective public health care measures.

## INTRODUCTION

Smoking is one of the main preventable causes of early development of diseases and death in the world. Smoking increases morbidity and mortality due to cardiovascular diseases, several types of cancer, and pulmonary diseases^16^. Children who live with smoking parents or who are exposed to the environmental smoke of tobacco have increased risk of asthma, acute respiratory diseases, respiratory symptoms such as coughing and wheezing, and middle ear infections compared with children in smoke-free environments^17^.

Smoking is usually started during adolescence. In the United States, most youngsters start smoking before the age of 18^22^. In Brazil, according to National School Health Survey (PeNSE), a study conducted with ninth graders, over 30.0% of 13- to 15-year-olds try smoking before the age of 12^1^. Nicotine addiction is rapidly established and young tobacco users are highly likely to keep smoking during their adult life. Early tobacco use is an independent predictor of the onset of nicotine addiction^15^.

Smoking is also one of the main causes of health inequality worldwide. Socioeconomically underprivileged children are more likely to engage in hazardous behaviors and to consequently have smoke-related illnesses^12^.

In order to deal with the increasing tobacco epidemic, member countries of the World Health Organization (WHO) developed the Framework Convention on Tobacco Control (FCTC), the first and only international public health treaty in the world, which has been in effect since February 2005^21^. Brazil ratified FCTC in November 2005, but had already implemented a series of comprehensive and effective measures for controlling tobacco use with the creation of its *Programa Nacional de Controle do Tabagismo* (PNCT – National Tobacco Control Program). These measures included advertising bans on all type of media; bans on misleading descriptions such as light, ultra light, and regular; a ban on smoking in public indoor places; mandatory warnings in cigarette packs; and, more recently, a consistent policy for increasing taxes and prices of tobacco products^a^.

As a consequence of such policy, the prevalence of tobacco use decreased considerably, going from 34.8% in 1989 to 14.7% in 2013 in the population of 18 years of age or older (including young adults – 18 to 24 years, going from 29.0% to 10.6%). However, Brazil still faces many challenges in that field, such as the need to prevent people from engaging in smoking and to avoid the problems caused by the higher prevalence of tobacco use among low-income youngsters and adults^14^.

This study aimed to estimate the prevalences of tobacco use, tobacco experimentation, and frequent smoking among Brazilian adolescents.

## METHODS

The Study of Cardiovascular Risks in Adolescents (ERICA) is a school based cross-sectional study that evaluated 12- to 17-year-old adolescents living in municipalities with over 100 thousand inhabitants in 2013/2014.

The sampling strategy of ERICA consisted in dividing the five regions into strata, which corresponded to state capital municipality and the set of municipalities with 100 thousand inhabitants or more in every region, totaling 32 strata. Following that, probabilistic sampling of schools was conducted in two stages. In the first one, the larger a school was, the higher its chances of being selected were. In turn, the farther away from a state capital a school was, the fewer chances it had of being picked. In the second one, three groups were selected from each unit that had been selected in the first stage. By using class years as variables for calculating ages, the only groups considered eligible were the ones in the seventh, eighth, and ninth grades of secondary school and the ones in the first, second, and third grades of high school. A detailed description of the sampling design can be found in a previous publication^18^. We selected 1,251 schools in 124 Brazilian municipalities. All students in the selected groups were invited to take part in the study. Adolescents of ages outside the eligible range, pregnant students, or physically-challenged ones were excluded from the analysis.

The information was obtained from self-administered questionnaires with eleven theme sections. They were applied in the classrooms under the supervision of the study team. Details on the study protocol may be seen in Bloch et al.^2^ All adolescents answered questions about smoking, including consumption intensity and characteristics and environmental exposure to tobacco fumes. An LG^®^ GM750Q handheld computer was used to collect the data.

In this study, tobacco experimentation was defined as having tried or smoked cigarettes at least once in life, even if one has only inhaled the smoke once or twice. The youngsters who had smoked cigarettes at least a day over the previous 30 days were considered current cigarette smokers. Both variables complied with the definitions by the World Health Organization (WHO) and by the Center for Disease Control and Prevention (CDC) in the Global Youth Tobacco Surveillance (GYTS)^20^. In order to investigate frequent tobacco use, information that is hard to obtain for this age range, having smoked cigarettes for at least seven consecutive days was used as an indicator.

For the number of smokers, we used population estimates obtained by processing microdata from the Demographic Sensuses 2000 and 2010 performed by the Brazilian Institute of Geography and Statistics (IBGE), which were used to define sampling fractions^b^. Estimates for the number of smokers and prevalence of variables regarding tobacco use were estimated with their respective 95% confidence intervals (95%CI) for the aggregate population, according to Brazilian region, sex, and age range (12-14 years; 15-17 years). We also estimated the percentages of current smokers according to categories of sociodemographic variables, as follows: ethnicity (black or *parda* ^c^ *versus* white or other), having had a paid job over the previous year (yes; no), type of family (living with both parents *versus* living only with the father, only with the mother, with no parents), father’s education level (up to seven years of schooling; eight years or longer), mother’s education level (up to seven years of schooling; eight years or longer). In regards to the socioenvironmental variables, the percentage of current tobacco use was estimated among adolescents who had been in contact with smokers at home (yes; no), and who had been in contact with smokers outside home (yes; no), and by type of school (public or private).

The data were analyzed by the routines for analysis of complex samples of Stata version 13.0^d^.

The study was approved by the Research Ethics Committee (REC) of the Instituto de Estudos em Saúde Coletiva of the Universidade Federal do Rio de Janeiro (IESC*/*UFRJ) and by a REC from each Brazilian state. Adolescents who signed assent forms took part in the study. Depending on state requirements, the adolescents had their parents sign informed consent forms.

## RESULTS

Among the 74,589 adolescents who took part in the study, 18.5% had smoked at least once in life; 5.7% smoked at the time of the research; and 2.5% had smoked for seven consecutive days (Table 1). The prevalences did not differ significantly between sexes. Regardless of sex, the percentage of use at least once in life was twice as high in the 15- to 17-year age range than in the 12- to 14-year one, almost twice as high for current tobacco use, and three times as high for use on over seven days in a row.

**Table 1:** Estimated number of smokers and prevalences of tobacco use among students according to consumption level, sex, age range, and macro-region. ERICA, Brazil, 2013-2014.

Variable	n_est_^a^	Brazil		North		Northeast		Midwest		Southeast		Sout	
					
		%	95%CI	%	95%CI	%	95%CI	%	95%CI	%	95%CI	%	95%CI
Tobacco experimentation^b^													
Male	956,348	18.8	17.7-19.9	20.2	18.9-21.4	15.7	13.7-18.0	21.8	18.9-25.2	18.5	16.8-20.3	22.4	20.0-25.1
12-14	284,045	10.5	9.4-11.7	11.6	10.2-13.1	8.9	6.6-12.0	12.0	10.1-14.2	9.1	7.6-11.0	17.8	14.6-21.5
15-17	672,303	28.0	26.4-29.8	29.7	27.7-31.7	23.3	20.3-26.5	32.8	27.2-38.9	29.1	26.4-32.0	27.8	24.9-30.9
Female	924,018	18.3	17.2-19.4	18.2	16.8-19.8	14.6	13.3-16.0	21.1	19.3-23.1	18.1	16.1-20.1	24.3	21.3-27.4
12-14	294,112	11.1	9.9-12.3	12.3	10.8-14.1	9.4	7.6-11.4	13.0	11.0-15.2	9.7	7.9-11.8	18.3	14.6-22.7
15-17	629,906	26.2	24.4-28.2	24.8	22.8-26.9	20.3	18.5-22.1	27.4	24.0-31.0	31.0	27.6-34.7	30.0	26.3-33.9

Total	1,880,367	18.5	17.7-19.4	19.2	18.1-20.4	15.2	13.9-16.5	21.5	19.9-23.1	18.3	16.9-19.8	23.3	21.5-25.3

Current tobacco use^c^													
Male	310,039	6.0	5.5-6.7	6.6	5.6-7.6	5.1	4.0-6.2	6.9	5.3-8.5	6.1	5.1-7.1	7.0	5.1-8.9
12-14	105,006	3.9	3.2-4.6	4.0	3.1-5.0	2.4	1.5-3.3	4.0	2.4-5.7	4.1	2.8-5.3	5.6	3.7-7.5
15-17	205,033	8.5	7.5-9.6	9.4	7.6-11.2	8.1	6.2-10.0	10.2	7.3 - 13.0	8.3	6.7-10.0	8.7	5.6-11.7
Female	269,024	5.3	4.8-5.8	5.1	4.3-6.1	4.2	3.4-5.1	5.7	4.6-6.8	5.2	4.5-6.0	7.6	5.7-9.5
12-14	98,965	3.7	3.1-4.3	3.8	2.9-4.8	3.4	2.2-4.5	4.2	2.8-5.6	3.2	2.3-4.2	6.1	3.9-8.3
15-17	170,059	7.1	6.3-7.9	6.7	5.4-8.0	5.2	4.4-6.1	7.3	5.9-8.8	7.4	6.0-8.8	9.3	6.3-12.2

Total	579,063	5.7	5.3-6.2	5.9	5.2-6.7	4.7	4.0-5.6	6.3	5.2-7.6	5.7	5.0-6.4	7.3	6.2-8.7

Smoked for seven consecutive days^d^													
Male	136066	2.7	2.3-3.1	2.3	1.9-2.9	1.7	1.1-2.4	4.5	3.0-6.9	2.7	2.1-3.5	3.3	2.6-4.4
12-14	29,515	1.1	0.8-1.4	0.8	0.5-1.3	1.1	0.5-2.2	1.6	1.0-2.6	0.8	0.5-1.4	2.1	1.2-3.8
15-17	106,551	4.4	3.7-5.3	4.0	3.1-5.1	2.3	1.7-3.2	7.8	4.7-12.7	4.8	3.7-6.3	4.8	3.7-6.1
Female	113,968	2.3	1.9-2.7	2.0	1.4-2.9	1.2	0.8-1.8	3.0	2.4-3.7	2.3	1.7-3.0	3.7	2.7-5.1
12-14	29,758	1.1	0.9-1.4	1.6	1.0-2.7	0.8	0.4-1.6	1.6	0.9-2.9	1.0	0.7-1.5	1.5	0.9-2.3
15-17	84,210	3.5	2.8-4.2	2.5	1.7-3.5	1.7	1.7-2.4	4.5	3.5-5.8	3.7	2.6-5.1	6.3	4.3-8.9

Total	250,034	2.5	2.2-2.8	2.2	1.7-2.7	1.5	1.1-1.9	2.5	1.9-3.1	3.5	2.9-4.3	3.8	2.8-5.1

^a^ n_est_: estimated number of smokers using population estimates based on processing of microdata from IBGE’s 2000 and 2010 Demographic Sensuses^b^.

^b^ Tobacco experimentation – tried smoking cigarettes at least once in life.

^c^ Current tobacco use – smoked at least one day over the previous 30 days.

^d^ Smoked for seven consecutive days – smoked for at least seven consecutive days in life.

Analysis of the regional pattern showed that higher prevalences for tobacco experimentation, current tobacco use, and tobacco use for seven days in a row were observed in the South region, especially when compared with the ones in the North and Northeast regions, which had the lowest prevalences. A similar profile was observed in both sexes for the three indicators.

Although the predominance of the male sex was only statistically significant for prevalences of tobacco experimentation among 15- to 17-year-old adolescents in the North region and for current tobacco use in the Northeast region, that was a trend observed in the specific estimates for the three indicators, in the total population and in the oldest age ranges. Exceptions to this pattern were observed in the South region, where women showed the highest percentages in all subgroups, and also in the Southeast region, where the female sex was also predominant in tobacco experimentation – but only in the oldest age range. However, we observed these differences only in specific estimates, without any statistical significance found. The youngest age group had very similar percentages for the male and female sexes.

Analyzing the sample by its strata (Figure), the prevalence of current tobacco use among men ranged from 2.8% in Boa Vista to 10.3% in Campo Grande. Vitoria, Cuiaba, Porto Alegre, and Florianopolis showed the highest percentages (≅ 7.5%) after Campo Grande. Following Boa Vista, the state capital municipalities with the lowest prevalences belong in the Northeast region, namely Salvador, Natal, Teresina, and Aracaju.

**Figure:**
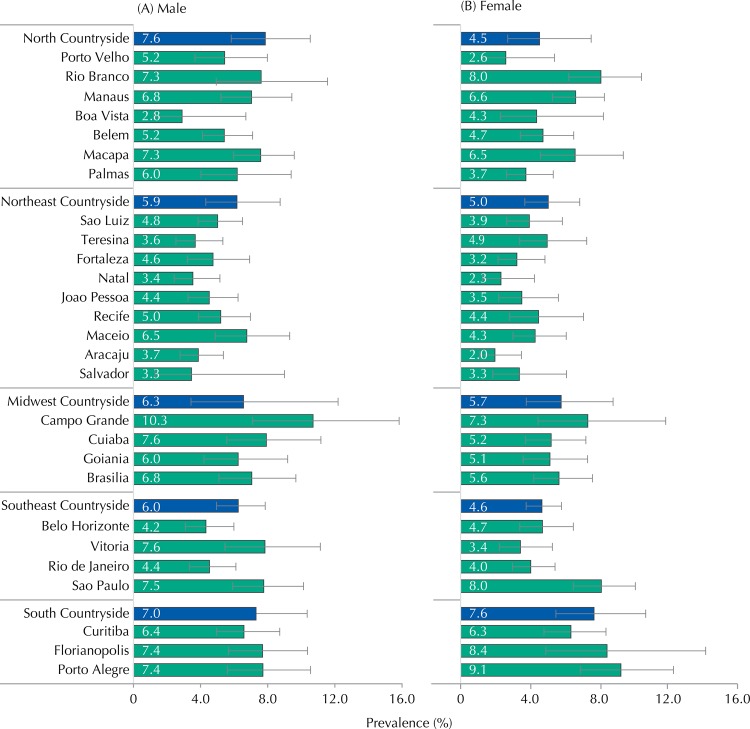
Prevalences (%) and 95%CI of tobacco use in male (A) and female (B) adolescents according to state capitals and strata of municipalities with over 100 thousand inhabitants in the countryside cities of the regions. ERICA, Brazil, 2013-2014.

Among the female adolescents, the prevalence of current tobacco use per geographical strata ranged from 2.0% to 9.1%. Higher percentages were observed in Porto Alegre, followed by Florianopolis, Sao Paulo, and Rio Branco, which showed averages from 8.0% on. Although the differences are not statistically significant, these cities consistently showed slightly higher percentages in the female sex than in the male one. Such as in the male sex, lower prevalences were also found in municipalities in the Northeast region (Aracaju and Natal) and in the North region (Porto Velho) (Figure).

The analysis of socioeconomic indicators (Table 2) shows there were no statistically significant differences in the prevalences of tobacco use according to reported ethnicity, education level, mother’s education level, and father’s education level for both sexes. Regardless of sex, the prevalence was higher for adolescents who had had paid jobs in the year prior to the study. Regarding the socioenvironmental indicators, higher prevalences were observed for youngsters who: did not live with both their parents, compared with the ones who did; reported having been in contact with smokers at home, compared with the ones who did not; had been in contact with smokers outside home, compared with the ones who did not. Female public school adolescents reported smoking more than the ones from private schools. Among males, that difference was only observed for specific estimates.

**Table 2:** Prevalence of tobacco use (% of students who smoked at least one day over the previous 30 days), according to socioeconomic variables, exposure to smokers, and sex. ERICA, Brazil, 2013-2014.

Variable	Male		Female	
	
	%	95%CI	%	95%CI
Ethnicity				
Black or *parda* ^a^	5.4	4.5-6.2	5.0	4.2-5.8
White or others	6.4	5.6-7.2	5.5	4.9-6.1
Mother’s education level (years of schooling)^b^				
< 8	5.6	4.9-6.2	4.6	4.0-5.3
≥ 8	5.7	4.3-7.1	5.4	4.4-6.4
Father’s education level (years of schooling)^c^				
< 8	4.9	3.9-5.8	3.1	2.4-4.1
≥ 8	4.9	3.3-6.4	4.3	2.7-5.9
Has had a paid job				
Yes	9.3	8.1-10.5	8.8	7.5-10.1
No	5.0	4.3-5.6	4.6	4.0-5.1
Type of family				
Living with both parents	4.8	4.0-5.5	4.4	3.8-5.1
Other	8.0	6.8-9.2	6.4	5.7-7.1
Has been in contact with smokers at home				
Yes	8.1	6.7-9.6	7.1	6.2-8.0
No	5.4	4.7-6.1	4.5	4.0-5.1
Has been in contact with smokers outside home				
Yes	9.9	8.6-11.1	7.6	6.8-8.4
No	3.6	3.0-4.3	2.7	2.2-3.3
Type of school				
Public	6.1	5.6-6.9	5.7	5.1-6.2
Private	5.2	3.5-7.0	3.7	2.3-5.1

^a^ Mixed race involving African ancestry.

^b^ 23.8% without information.

^c^ 24.4% without information.

## DISCUSSION

As ERICA is a large-scale study with a sample representative of a population of adolescent students of a wide age range (12 to 17 years) from municipalities with over 100 thousand inhabitants, its results provide a unique opportunity to evaluate the extent of tobacco use in this population. The study shows that 1.88 million adolescents of the target ages of the study reported having tried smoking, 579 thousand of them currently smoke, and 250 thousand have already smoked somewhat regularly, and that is a considerable number of youngsters with high chances of becoming regular smokers and addicted to nicotine^9^.

By comparing the results from ERICA with the ones from the PeNSE conducted in 2012, the prevalences of tobacco experimentation (18.5% *versus* 19.6%, respectively) and current tobacco use (5.7% *versus* 5.1%, respectively) were similar, despite the target population of PeNSE being younger (86,0% between 13 and 15 years)^6^.

In American countries that conducted the Global Youth Tobacco Survey, in different years between 2000 and 2010, the percentage of current cigarette smokers in the age range between 13 and 15 years varied considerably^e^. In the set of Latin American surveys, the average found was 16.3%. The lowest average – 4.3% – was found in Panama, and the highest one – 34.2% – in Chile, both in 2008. With the exception of Venezuela and Paraguay, all South American countries showed percentages above 15.0%. Therefore, prevalences found in Brazil are low when compared with its neighbor countries, and that is possibly the result from the country’s pioneer conduction of comprehensive, high-impact policies to reduce tobacco consumption among youngsters. Among these are the legislation that banned smoking in public indoor environments, the ban on advertising in all media, with the exception of points of sale, and the ban on misleading descriptions such as light or ultra light in cigarette packs^8^.

The prevalences for consumption of cigarettes from the *Pesquisa Nacional de Saúde* (PNS – Brazilian National Health Survey), conducted in 2013 with 18- to 24-year-old subjects, found higher shares than the ones observed in ERICA’s 15- to 17-year-olds (mainly among boys). That suggests ERICA has not reflected the whole initiation as it is a process that, in Brazil, is also observed among young adults. One may point out as positive the fact that this population group also quits smoking more, which has been decreasing the share of 18- to 24-year-old smokers with time^10^ ^,^ ^14^.

Among youngsters, the prevalence of tobacco use almost always increases with age, but the results found show a rapid evolution of tobacco use already at early ages. That information reinforces the importance of maintaining and expanding effective policies for this age group, aiming to reduce tobacco experimentation and its transition into regular use of cigarettes with consequent establishment of nicotine addiction. Besides the increase with age, world and Brazilian literature consistently show higher prevalences of tobacco use among men than among women^16^. The pattern observed among adolescents is variable. Higher percentages are more common in the male sex; however, in regions and countries with high economic development, such as most countries in Europe and the United States, young female shows higher prevalence^19^. The causes of differences between sexes are complex and multi-factorial.

The regional profile of tobacco use in ERICA is very similar to the one in PeNSE, with higher percentages of tobacco experimentation and current smoking in the South and Midwest regions, intermediate values in the Southeast region, and smaller values in the Northeast region. That profile of high prevalence in the South region has been observed both in household surveys targeting adults and among students^10^ ^,^ ^f^. As in ERICA, in these studies the women from the South region stand out even further when compared with the ones from other regions.

Factors associated with this higher prevalence of initiation in the South region, especially Porto Alegre, in relation to the remaining regions, must be investigated. Among the hypothesis proposed, we mention the concentration of tobacco crops in this region. The tobacco industry performs activities of different natures to convince authorities and the population of its economic importance, such as sponsoring community events and institutions, promoting a positive self-image, which could be seen as indirect advertising^g^. An alternative explanation could be the higher influence from European immigrants, who traditionally have high prevalences and transfer such influence throughout generations. The finding of a lower prevalence for the Northeast region, regardless of sex, has not been clarified either. The regional pattern reflects that observed in the state capitals, in which higher percentages were observed in the cities of the South region, with highlights to the female sex and to Campo Grande.

Strong scientific evidence indicates that, in the world and in Brazil, active and passive tobacco use is concentrated in low-income populations, which show more frequent tobacco initiation than population groups with higher purchasing power^5^ ^,^ ^7^ ^,^ ^11^. Social, environmental, and individual conditions are pointed out in order to explain the higher vulnerability of poorer people. Among those, David et al.^4^ mention underprivileged adolescents’ poorer skills to resist the pressure from their parents and friends and from tobacco advertising, as a consequence of their weaker social skills, self-confidence, and self-esteem; higher prevalence among parents and friends, which fosters initiation among the poor; higher social stress; higher prevalence of psychiatric and alcohol-related comorbidities; and less social support.

In ERICA, the only indicators of socioeconomic conditions found to have strong ties with tobacco use were the type of family and having worked over the previous year. The relationship with the type of family was observed in previous studies^1^ ^,^ ^11^. It is suggested that children who live with both their parents have better emotional structure and supervision from their parents in order to reinforce abstinence at this age. The importance of parental supervision was also observed in PeNSE, in which indicators such as “parents would care if their child smoked” were associated with the lower frequency of risk behaviors^6^.

The association with work can be explained by two factors. Youngsters of that age generally work because they need to, and, therefore, they have low socioeconomic statuses; furthermore, these adolescents have enough income to buy cigarettes. This combination represents an important factor that drives consumption, and that shows the importance of measures to increase prices and taxes to reduce the use of tobacco in the general population, especially among young people^3^. The potential of those measures was suggested by Szklo et al.^14^, which link the reduction in the number of 18- to 24-year-old smokers – observed in PNS – to the raise in cigarette prices in recent years.^a^

Unlike other studies conducted in Brazil, ERICA did not show tobacco use and parents’ education level were inversely associated^1^ ^,^ ^6^. Classification errors could be used as a hypothesis to back such finding, based on the fact that some adolescents do not know what their parents’ education levels are. Almost 1/5 of the adolescents in ERICA reported not knowing or remembering their parents’ education levels.

Attending public schools is expected to be a proxy for low socioeconomic status and a social environment that, for all previously mentioned aspects related to poverty, could be associated with smoking. Such expected pattern was observed among female adolescents. However, in the male sex, the existing difference was not significant. Deeper analyses, which account for possible confounding factors and effect modification, may better clarify such relationships.

The association with having been in the presence of smokers at home or outside home corroborates the literature^15^ ^,^ ^17^. For young people, the policies for promoting smoke-free environments are justified by the importance of “denormalization” and consequently by a community’s lower acceptance of the smoking behavior^13^. In 2012, Brazil regulated its law of smoke-free environments, fully banning the habit of smoking from closed public environments^h^. The potential of this law was pointed out in a recent study, in which a significant reduction in the exposure to passive smoking at home and at work was observed among the Brazilians, including the younger ones^14^.

The absolute number and the prevalence of smokers observed in this study show that tobacco use among adolescents is still a challenge that requires effective public health care measures. Aiming to reduce the prevalence of tobacco use among young people, especially the ones under socioeconomic vulnerability conditions, Brazil must consolidate and increase the measures provided in the Framework Convention on Tobacco Control, which have great impacts in this population subgroup. Among these, we highlight the need to maintain and strengthen policies for raising taxes and prices and for fully banning advertising in places that sell tobacco products.

## Footnotes

a Instituto Nacional de Câncer (INCA). Observatório da Política Nacional de Controle do Tabaco. Rio de Janeiro: Instituto Nacional do Câncer; 2015 [cited 2015 Sept 30]. Available from: http://www2.inca.gov.br/wps/wcm/connect/observatorio_controle_tabaco/site/home

b Instituto Brasileiro de Geografia e Estatística. Projeção da população no Brasil por sexo e idade: 2000-2060. Rio de Janeiro; 2013 [cited 2015 Sept]. Available from: http://www.ibge.gov.br/home/estatistica/populacao/projecao_da_populacao/2013/default.shtm

c Mixed race involving African ancestry.

d StataCorp. Stata Statistical Software: Release 14. College Station, TX: StataCorp LP; 2015.

e Panamerican Health Organization. Youth and tobacco in the Americas: results from the Global Youth Tobacco Survey 2000-2010. Washington (DC); Panamerican Health Organization [cited 2015 Sept 18]. Available from: http://www.paho.org/hq./index.php?option=com_docman&task=doc_view&gid=17483&Itemid=3482&lang=pt

f Instituto Nacional de Câncer – INCA. Vigescola. Vigilância de Tabagismo em Escolares. Dados e fatos de 17 cidades brasileiras. Instituto Nacional de Câncer, Rio de Janeiro, 2009. Available from: http://www.inca.gov.br/vigescola/

g Instituto Nacional de Câncer (INCA). O programa de controle do tabagismo no Brasil: avanços e desafios. Rio de Janeiro: Instituto Nacional do Câncer; 2006.

h Brasil. Decreto nº 8.262, de 31 de maio de 2014. Altera o Decreto nº 2.018, de 1º de outubro de 1996, que regulamenta a Lei nº 9.294, de 15 de julho de 1996. *Diario Oficial Uniao*, Brasília (DF), 2 junho, 2014. Seção 1, p. 108
