# Supplementary material for: ERICA: smoking prevalence in Brazilian adolescents
Source: Rev Saude Publica. 2016 Feb 2;50(Suppl 1):12s. doi: 10.1590/S01518-8787.2016050006741 (PMC4767033; doi:10.1590/S01518-8787.2016050006741)
Supplement: Translation

# Translation: ERICA: prevalência de tabagismo em adolescentes brasileiros

**Authors:** Valeska Carvalho Figueiredo, André Salem Szklo, Letícia Casado Costa, Maria Cristina C Kuschnir, Thiago Luiz Nogueira da Silva, Katia Vergetti Bloch, Moyses Szklo

**Affiliations:** I Centro de Estudos sobre Tabaco e Saúde. Escola Nacional de Saúde Pública Sergio Arouca. Fundação Oswaldo Cruz. Rio de Janeiro, RJ, Brasil; II Divisão de Epidemiologia. Coordenação Geral de Ensino e Pesquisa. Instituto Nacional de Câncer José Alencar Gomes da Silva. Rio de Janeiro, RJ, Brasil; III Serviço de Edição e Informação Técnico Científica. Coordenação de Prevenção e Vigilância. Instituto Nacional de Câncer José Alencar Gomes da Silva. Rio de Janeiro, RJ, Brasil; IV Faculdade de Ciências Médicas. Núcleo de Estudos da Saúde do Adolescente. Universidade do Estado do Rio de Janeiro. Rio de Janeiro, RJ, Brasil; V Instituto de Estudos em Saúde Coletiva. Universidade Federal do Rio de Janeiro. Rio de Janeiro, RJ, Brasil

**Keywords:** Adolescente, Tabagismo, Epidemiologia, Prevalência, Inquérito

## Abstract

**OBJETIVO:**

Estimar as prevalências de tabagismo, experimentação e fumo frequente em adolescentes brasileiros.

**MÉTODOS:**

Foram avaliados os participantes do Estudo de Riscos Cardiovasculares em Adolescentes (ERICA), estudo transversal de base escolar e abrangência nacional. Participaram adolescentes de 12 a 17 anos de municípios com mais de 100 mil habitantes. A amostra foi estratificada e conglomerada e tem representatividade nacional, regional e para as 27 capitais. As informações foram obtidas usando-se questionário autopreenchível. Experimentação foi definida como: ter experimentado cigarros alguma vez na vida. Foram considerados fumantes atuais de cigarros aqueles que fumaram pelo menos um dia nos últimos 30 dias. Utilizou-se como indicador de uso frequente de tabaco ter fumado cigarros por pelo menos sete dias seguidos. Considerando-se o desenho complexo da amostra, prevalências e intervalos de confiança de 95% foram estimados segundo características sociodemográficas e socioambientais.

**RESULTADOS:**

Foram avaliados 74.589 adolescentes; dentre esses, 18,5% (IC95% 17,7-19,4) fumaram pelo menos uma vez na vida, 5,7% (IC95% 5,3-6,2) fumavam no momento da pesquisa e 2,5% (IC95% 2,2-2,8) fumavam com frequência. Adolescentes de 15 a 17 anos tiveram prevalências mais elevadas de todos os indicadores comparados aos de 12 a 14 anos. As prevalências não apresentaram diferenças significativas entre sexos. Maiores prevalências foram observadas na região Sul e menores na região Nordeste. Independentemente de sexo, as prevalências foram maiores para adolescentes que tinham tido trabalho remunerado, nos que não moravam com os dois pais e que referiram ter tido contato com fumante em casa ou fora. Adolescentes do sexo feminino de escolas públicas fumavam mais do que as de escolas privadas.

**CONCLUSÕES:**

O tabagismo entre adolescentes ainda é um desafio. Visando a redução da prevalência de tabagismo entre jovens, em especial os que se encontram em situação de vulnerabilidade socioeconômica, o Brasil deve consolidar e ampliar medidas de saúde pública efetivas.

## INTRODUÇÃO

O tabagismo é uma das principais causas evitáveis de adoecimento e morte precoces do mundo. Fumar aumenta a morbimortalidade por doenças cardiovasculares, vários tipos de câncer e doenças pulmonares^16^. Crianças que convivem com pais fumantes ou que estão expostas à fumaça ambiental do tabaco apresentam maior risco de episódios de asma, doença respiratória aguda, sintomas respiratórios como tosse e chiado e infecções de ouvido médio, do que crianças não expostas^17^.

O início do tabagismo em geral ocorre na adolescência. Nos Estados Unidos, a grande maioria dos jovens começa a fumar antes dos 18 anos de idade^22^. No Brasil, segundo dados da Pesquisa Nacional de Saúde do Escolar (PeNSE), realizada com estudantes do nono ano, mais de 30,0% dos jovens de 13 a 15 anos experimenta fumar antes dos 12 anos de idade^1^. A dependência da nicotina se estabelece rapidamente e a probabilidade de jovens usuários de tabaco continuarem fumando na fase adulta é elevada. O início precoce do tabagismo é um preditor independente da instalação da dependência da nicotina^15^.

O tabagismo é também uma das principais causas de desigualdade em saúde no mundo. Crianças em desvantagem socioeconômica têm maior probabilidade de se engajarem em comportamentos de risco e, consequentemente, de desenvolverem agravos associados ao fumo^12^.

Para enfrentar a escalada da epidemia do tabaco, países membros da Organização Mundial da Saúde (OMS) desenvolveram a Convenção Quadro Para o Controle do Tabaco (CQCT), primeiro e único tratado internacional de saúde pública do mundo, o qual se encontra em vigor desde fevereiro de 2005^21^. O Brasil ratificou a CQCT em novembro de 2005, mas já em 1989, com a criação do Programa Nacional de Controle do Tabagismo (PNCT), implementou uma série de medidas abrangentes e efetivas de controle do tabaco, tais como: proibição da propaganda em todos os tipos de mídia; proibição de descritores enganosos *light, ultra-light* e regular; proibição de fumar em ambientes públicos fechados; obrigatoriedade na implantação de advertências com imagens nos maços de cigarros; e, mais recentemente, uma política consistente de aumento de impostos e preços dos produtos do tabaco^a^.

Como consequência dessa política, a prevalência de tabagismo diminuiu consideravelmente, passando de 34,8% em 1989 a 14,7% em 2013 na população de 18 anos ou mais (inclusive entre os jovens adultos – 18 a 24 anos, passando de 29,0% a 10,6%). Porém o País ainda enfrenta muitos desafios nesse campo, como a necessidade de reduzir a iniciação e as inequidades provocadas pela maior prevalência de tabagismo em jovens e adultos de baixa renda^14^.

O objetivo deste estudo foi estimar as prevalências de prevalências de tabagismo, experimentação de cigarros e fumo frequente em adolescentes brasileiros.

## MÉTODOS

O Estudo de Riscos Cardiovasculares em Adolescentes (ERICA) consiste em um estudo transversal, de base escolar, realizado em 2013-2014, em amostra representativa de escolares de 12 a 17 anos de municípios brasileiros com mais de 100 mil habitantes.

A estratégia de amostragem do ERICA consistiu em dividir as regiões em estratos correspondentes aos municípios das capitais e ao conjunto de municípios com 100 mil habitantes ou mais pertencentes ao interior de cada região, em um total de 32 estratos. Em seguida, realizou-se uma amostragem probabilística de escolas em dois estágios. No primeiro, foram selecionadas escolas com probabilidade diretamente proporcional ao tamanho das mesmas e inversamente proporcional à distância da capital. No segundo, selecionaram-se três turmas em cada unidade selecionada no primeiro estágio. Usando-se o ano da turma como variável informativa da idade, considerou-se elegíveis para a seleção apenas as classes dos sétimo, oitavo e nono anos do ensino fundamental e primeiro, segundo e terceiro anos do ensino médio. Descrição detalhada do desenho amostral encontra-se em publicação anterior^18^. Foram selecionadas 1.251 escolas em 124 municípios brasileiros. Todos os escolares das turmas selecionadas foram convidados a participar do estudo. Adolescentes fora da faixa etária elegível, grávidas ou com alguma deficiência física foram excluídos da análise.

As informações foram obtidas a partir de um questionário autopreenchível, compreendendo onze blocos temáticos, aplicado em salas de aula, sob supervisão da equipe do estudo. Em Bloch et al.^2^ podem ser vistos detalhes sobre o protocolo. Todos os jovens responderam a perguntas sobre tabagismo, incluindo questões sobre intensidade e características do consumo e exposição ambiental à fumaça do tabaco. Para a coleta de dados utilizou-se um coletor eletrônico de dados individual portátil, modelo LG^®^ GM750Q.

Neste estudo, definiu-se como experimentação ter provado ou fumado cigarros alguma vez na vida, mesmo uma ou duas tragadas. Foram considerados fumantes atuais de cigarros os jovens que fumaram cigarros pelo menos um dia nos últimos 30 dias. Ambas as variáveis seguiram as definições adotadas pela OMS e pelo Centro para Prevenção e Controle de Doenças dos Estados Unidos (*Centers for Disease Control and Prevention* – CDC) na Vigilância Mundial de Tabagismo em Jovens (*Global Youth Tobacco Surveillance* – GYTS)^20^. Para investigar o uso frequente de tabaco, informação de difícil apreensão para esse grupo etário, utilizou-se como indicador ter fumado cigarros por pelo menos sete dias seguidos.

O número de fumantes foi calculado utilizando-se estimativas de população obtidas por meio de processamento dos microdados dos Censos Demográficos de 2000 e 2010 do IBGE, cujas informações foram usadas para a definição de frações de amostragem^b^. Estimativas do número de fumantes e a prevalência das variáveis relativas ao tabagismo com seus respectivos intervalos de 95% de confiança (IC95%) foram calculadas para a população agregada, por regiões brasileiras, sexo e faixa etária (12-14 anos e 15-17 anos). Foram ainda calculados os percentuais de fumantes atuais segundo categorias de variáveis sociodemográficas, sendo essas: cor da pele (preta ou parda *versus* branca ou outra), ter tido trabalho remunerado no último ano (sim ou não), tipo de família (mora com pai e mãe *versus* mora somente com pai, somente com mãe, não mora com nenhum dos pais), escolaridade do pai (até sete anos de estudo *versus* oito anos ou mais), escolaridade da mãe (até sete anos de estudo *versus* oito anos ou mais). Quanto às variáveis socioambientais, estimou-se o percentual de tabagismo atual entre os adolescentes que estiveram em contato com fumantes em casa (sim ou não), que estiveram em contato com fumantes fora de casa (sim ou não) e por tipo de escola (pública ou privada).

Os dados foram analisados utilizando-se rotinas para análise de amostras complexas do Stata, versão 13.0^c^.

O estudo foi aprovado pelo Comitê de Ética em Pesquisa (CEP) do Instituto de Estudos em Saúde Coletiva da Universidade Federal do Rio de Janeiro (IESC/UFRJ) e por um CEP de cada unidade da federação. Participaram do estudo os adolescentes que assinaram termo de assentimento e, quando exigido pelo CEP local, eles trouxeram termo de consentimento livre e esclarecido assinado pelo responsável.

## RESULTADOS

Dos 74.589 adolescentes que participaram do estudo, 18,5% fumaram pelo menos uma vez na vida, 5,7% fumavam no momento da pesquisa e 2,5% havia fumado por sete dias seguidos (Tabela 1). As prevalências não apresentaram diferenças significativas entre sexos. Independentemente do sexo, o percentual de uso pelo menos uma vez na vida foi mais que o dobro na faixa etária de 15 a 17 anos em relação à de 12 a 14 anos, quase o dobro para tabagismo atual e três vezes maior para uso em mais de sete dias seguidos.

**Tabela 1:** Número estimado de escolares fumantes e prevalências de tabagismo por nível de consumo, sexo, grupo etário e macrorregião. ERICA, Brasil, 2013-2014.

Variável	n_est_^a^	Brasil		Norte		Nordeste		Centro-Oeste		Sudeste		Sul	
					
		%	IC95%	%	IC95%	%	IC95%	%	IC95%	%	IC95%	%	IC95%
Experimentação^b^													
Masculino	956.348	18,8	17,7-19,9	20,2	18,9-21,4	15,7	13,7-18,0	21,8	18,9-25,2	18,5	16,8-20,3	22,4	20,0-25,1
12-14	284.045	10,5	9.4-11.7	11,6	10.2-13.1	8,9	6,6-12,0	12,0	10,1-14,2	9,1	7,6-11,0	17,8	14,6-21,5
15-17	672.303	28,0	26,4-29,8	29,7	27,7-31,7	23,3	20,3-26,5	32,8	27,2-38,9	29,1	26,4-32,0	27,8	24,9-30,9
Feminino	924.018	18,3	17,2-19,4	18,2	16,8-19,8	14,6	13,3-16,0	21,1	19,3-23,1	18,1	16,1-20,1	24,3	21,3-27,4
12-14	294.112	11,1	9,9-12,3	12,3	10,8-14,1	9,4	7,6-11,4	13,0	11,0-15,2	9,7	7,9-11,8	18,3	14,6-22,7
15-17	629.906	26,2	24,4-28,2	24,8	22,8-26,9	20,3	18,5-22,1	27,4	24,0-31,0	31,0	27,6-34,7	30,0	26,3-33,9

Total	1.880.367	18,5	17,7-19,4	19,2	18,1-20,4	15,2	13,9-16,5	21,5	19,9-23,1	18,3	16,9-19,8	23,3	21,5-25,3

Tabagismo atual^c^													
Masculino	310.039	6,0	5,5-6,7	6,6	5,6-7,6	5,1	4,0-6,2	6,9	5,3-8,5	6,1	5,1-7,1	7,0	5,1-8,9
12-14	105.006	3,9	3,2-4,6	4,0	3,1-5,0	2,4	1,5-3,3	4,0	2,4-5,7	4,1	2,8-5,3	5,6	3,7-7,5
15-17	205.033	8,5	7,5-9,6	9,4	7,6-11,2	8,1	6,2-10,0	10,2	7,3-13,0	8,3	6,7-10,0	8,7	5,6-11,7
Feminino	269.024	5,3	4,8-5,8	5,1	4,3-6,1	4,2	3,4-5,1	5,7	4,6-6,8	5,2	4,5-6,0	7,6	5,7-9,5
12-14	98.965	3,7	3,1-4,3	3,8	2,9-4,8	3,4	2,2-4,5	4,2	2,8-5,6	3,2	2,3-4,2	6,1	3,9-8,3
15-17	170.059	7,1	6,3-7,9	6,7	5,4-8,0	5,2	4,4-6,1	7,3	5,9-8,8	7,4	6,0-8,8	9,3	6,3-12,2

Total	579.063	5,7	5,3-6,2	5,9	5,2-6,7	4,7	4,0-5,6	6,3	5,2-7,6	5,7	5,0-6,4	7,3	6,2-8,7

Fumou 7 dias seguidos^d^													
Masculino	136.066	2,7	2,3-3,1	2,3	1,9-2,9	1,7	1,1-2,4	4,5	3,0-6,9	2,7	2,1-3,5	3,3	2,6-4,4
12-14	29.515	1,1	0,8-1,4	0,8	0,5-1,3	1,1	0,5-2,2	1,6	1,0-2,6	0,8	0,5-1,4	2,1	1,2-3,8
15-17	106.551	4,4	3,7-5,3	4,0	3,1-5,1	2,3	1,7-3,2	7,8	4,7-12,7	4,8	3,7-6,3	4,8	3,7-6,1
Feminino	113.968	2,3	1,9-2,7	2,0	1,4-2,9	1,2	0,8-1,8	3,0	2,4-3,7	2,3	1,7-3,0	3,7	2,7-5,1
12-14	29.758	1,1	0,9-1,4	1,6	1,0-2,7	0,8	0,4-1,6	1,6	0,9-2,9	1,0	0,7-1,5	1,5	0,9-2,3
15-17	84.210	3,5	2,8-4,2	2,5	1,7-3,5	1,7	1,7-2,4	4,5	3,5-5,8	3,7	2,6-5,1	6,3	4,3-8,9

Total	250.034	2,5	2,2-2,8	2,2	1,7-2,7	1,5	1,1-1,9	2,5	1,9-3,1	3,5	2,9-4,3	3,8	2,8-5,1

^a^ n_est_: número estimado de fumantes utilizando-se estimativas de população obtidas por meio de processamento dos microdados dos Censos Demográficos de 2000 e 2010 do IBGE^b.^

^b^ Experimentação – tentou ou experimentou fumar cigarros alguma vez na vida.

^c^ Tabagismo atual – fumou pelo menos 1 dia nos últimos 30 dias.

^d^ Fumou 7 dias seguidos – fumou pelo menos 7 dias seguidos na vida.

A análise do padrão regional mostrou que maiores prevalências de experimentação, tabagismo atual e uso em sete dias seguidos foram observadas na região Sul, sobretudo quando comparadas às das regiões Norte e Nordeste, que apresentaram as prevalências mais baixas. Perfil semelhante foi observado para ambos os sexos para os três indicadores.

Embora o predomínio no sexo masculino tenha sido estatisticamente significante apenas para as prevalências de experimentação entre jovens de 15 a 17 anos no Norte e de tabagismo atual no Nordeste, essa foi uma tendência observada para as estimativas pontuais dos três indicadores, na população total e nas faixas etárias mais avançadas. Exceções a esse padrão ocorreram na região Sul, onde mulheres apresentaram maiores percentuais em todos os subgrupos, e na região Sudeste, onde também houve predomínio do sexo feminino para experimentação, mas apenas na faixa etária mais velha. Porém, essas diferenças também foram observadas apenas nas estimativas pontuais, sem que houvesse significância estatística. O grupo etário mais jovem apresentou percentuais muito semelhantes para o sexo masculino e feminino.

Analisando-se por estrato da amostra (Figura), a prevalência de tabagismo atual entre homens variou de 2,8% em Boa Vista a 10,3% em Campo Grande. Vitória, Cuiabá, Porto Alegre e Florianópolis apresentaram os maiores percentuais (≅ 7,5%) depois de Campo Grande. Seguindo-se Boa Vista, os municípios da capital com menores prevalências pertencem à região Nordeste, sendo estes Salvador, Natal, Teresina e Aracaju.

**Figura:** Prevalências (%) e IC95% de tabagismo em adolescentes do sexo masculino (A) e feminino (B) por capitais e estratos de municípios com mais de 100 mil habitantes do interior das regiões. ERICA, Brasil, 2013-2014.

Entre adolescentes do sexo feminino, a prevalência de tabagismo corrente segundo estrato geográfico variou de 2,0% a 9,1%. Maiores percentuais foram observados em Porto Alegre, seguido de Florianópolis, São Paulo e Rio Branco, que apresentaram médias a partir de 8,0%. Embora as diferenças não sejam estatisticamente significantes, essas cidades consistentemente apresentaram percentuais discretamente mais elevados no sexo feminino do que no masculino. Assim como no sexo masculino, menores prevalências foram observadas em municípios das regiões Nordeste (Aracaju e Natal) e Norte (Porto Velho) (Figura).

A análise de indicadores socioeconômicos (Tabela 2) mostra que não houve diferença estatisticamente significante nas prevalências de tabagismo de cigarros segundo cor da pele referida, nível de escolaridade da mãe e nível de escolaridade do pai para ambos os sexos. Independentemente de sexo, a prevalência foi maior para adolescentes que tinham tido trabalho remunerado no ano anterior ao estudo. Quanto aos indicadores socioambientais, prevalências mais elevadas foram observadas para jovens que: não moravam com os dois pais, em comparação aos que moravam com os dois pais; referiram ter tido contato com fumante em casa, em relação aos que referiram não ter tido contato com fumantes em casa; e tiveram contato com fumantes fora de casa, em comparação aos que não tiveram contato fora de casa. Adolescentes do sexo feminino de escolas públicas relataram fumar mais do que as de escolas privadas. No sexo masculino, essa diferença só foi observada para as estimativas pontuais.

**Tabela 2:** Prevalência (%) de tabagismo (% de escolares que fumou em pelo menos 1 dia nos últimos 30 dias), segundo variáveis socioeconômicas, exposição a fumantes e sexo. ERICA, Brasil, 2013-2014.

Variável	Masculino		Feminino	
	
	%	IC95%	%	IC95%
Cor da pele				
Preta ou parda	5,4	4,5-6,2	5,0	4,2-5,8
Branca ou outras	6,4	5,6-7,2	5,5	4,9-6,1
Escolaridade da mãe (anos de estudo)^a^				
< 8	5,6	4,9-6,2	4,6	4,0-5,3
≥ 8	5,7	4,3-7,1	5,4	4,4-6,4
Escolaridade do pai (anos de estudo)^b^				
< 8	4,9	3,9-5,8	3,1	2,4-4,1
≥ 8	4,9	3,3-6,4	4,3	2,7-5,9
Trabalhou remuneradamente				
Sim	9,3	8,1-10,5	8,8	7,5-10,1
Não	5,0	4,3-5,6	4,6	4,0-5,1
Tipo de família				
Mora com pai e mãe	4,8	4,0-5,5	4,4	3,8-5,1
Outra	8,0	6,8-9,2	6,4	5,7-7,1
Esteve em contato com fumante em casa				
Sim	8,1	6,7-9,6	7,1	6,2-8,0
Não	5,4	4,7-6,1	4,5	4,0-5,1
Esteve em contato com fumante fora de casa				
Sim	9,9	8,6-11,1	7,6	6,8-8,4
Não	3,6	3,0-4,3	2,7	2,2-3,3
Tipo de escola				
Pública	6,1	5,6-6,9	5,7	5,1-6,2
Privada	5,2	3,5-7,0	3,7	2,3-5,1

^a^ 23,8% sem informação.

^b^ 24,4% sem informação.

## DISCUSSÃO

Sendo o ERICA um estudo de larga escala com amostra representativa de uma população de adolescentes, estudantes, numa faixa ampla de idade (12 a 17 anos) de municípios com mais de 100 mil habitantes, seus resultados oferecem uma oportunidade singular para avaliar a magnitude do tabagismo nesta população. O estudo mostra que mais de 1,88 milhões de adolescentes nas idades alvo do estudo experimentou fumar, 579 mil fuma atualmente e 250 mil já fumou com alguma regularidade, um número considerável de jovens com elevada probabilidade de se tornarem fumantes regulares e de desenvolverem a dependência da nicotina^9^.

Confrontando-se os resultados do ERICA com os da PeNSE, realizada em 2012, a prevalência de experimentação (18,5% *versus* 19,6%, respectivamente) e de tabagismo atual (5,7% *versus* 5,1%, respectivamente) foram semelhantes, apesar de a população alvo da PeNSE ser mais jovem (86,0% entre 13-15 anos)^6^.

Em países das Américas que realizaram o *Global Youth Tobacco Survey*, em anos distintos compreendidos entre 2000 e 2010, o percentual de fumantes atuais de cigarros na faixa etária de 13 a 15 anos variou consideravelmente^d^. No conjunto de levantamentos feitos na América Latina, a média encontrada foi de 16,3%, a mais baixa de 4,3% no Panamá e a mais elevada de 34,2% no Chile, ambas em 2008. À exceção da Venezuela e Paraguai, todos os países da América do Sul apresentaram percentuais acima de 15,0%. No Brasil, portanto, encontram-se prevalências baixas quando comparado a países vizinhos, possivelmente um reflexo do pioneirismo do País na condução de políticas abrangentes e de grande impacto na redução do consumo de tabaco entre jovens. Entre essas, estão a legislação que instituiu a proibição de fumar em ambientes públicos fechados, a proibição da propaganda em todas as mídias exceto em pontos de venda e a proibição de descritores enganosos *light* ou *ultra-light* nos maços de cigarros^8^.

As prevalências de consumo de cigarro da Pesquisa Nacional de Saúde (PNS) realizada em 2013, com participantes de 18 a 24 anos, apresentam proporções mais altas do que aquelas observadas na população de 15 a 17 anos do ERICA (principalmente entre os meninos). Isso sugere que o ERICA não tenha capturado toda a iniciação por ser um processo que, no Brasil, acontece também entre adultos jovens. Pode-se apontar como um dado positivo o fato de que a cessação neste público jovem também aumentou, fazendo com que a proporção de fumantes entre 18-24 anos venha diminuindo ao longo do tempo^10^ ^,^ ^14^.

Entre jovens, a prevalência de tabagismo quase sempre aumenta com a idade; mas os resultados encontrados mostram evolução do tabagismo ainda em idades precoces. Esse dado reforça a importância da manutenção e expansão de políticas efetivas para este grupo etário visando a redução da experimentação e da transição desta para uso frequente de cigarros e consequente estabelecimento da dependência da nicotina. Além do aumento com a idade, a literatura mundial e do Brasil consistentemente mostra em adultos prevalências de tabagismo maiores entre homens do que entre mulheres^16^. O padrão observado entre adolescentes é variável. Percentuais mais elevados no sexo masculino são mais frequentes, porém em regiões e países com elevado nível de desenvolvimento econômico, como a maior parte dos países da Europa e Estados Unidos, a prevalência é mais alta no sexo feminino^19^. As causas das diferenças entre gêneros são complexas e mutifatoriais.

O perfil regional do tabagismo no ERICA é muito semelhante ao observado na PeNSE, com maiores percentuais de experimentação e fumo atual no Sul e Centro-Oeste, valores intermediários no Sudeste e Norte e menores valores no Nordeste. Esse padrão de prevalência elevada na região Sul tem sido observado tanto em estudos domiciliares com alvo em adultos, quanto entre escolares^10^ ^,^ ^e^. Assim como no ERICA, nesses estudos, as mulheres da região Sul destacam-se ainda mais quando comparadas a outras regiões.

Fatores associados a essa prevalência de iniciação mais elevada na região Sul, em especial Porto Alegre, do que nas demais regiões devem ser investigados. Entre as hipóteses levantadas, menciona-se o fato de esta região concentrar o cultivo e produção de fumo. A indústria do tabaco desenvolve atividades de diversas naturezas para convencer as autoridades e a população de sua importância econômica, tais como patrocínio de eventos e instituições comunitárias, promovendo uma autoimagem positiva, a qual poderia ser uma forma de propaganda indireta^f^. Uma explicação alternativa seria a maior influência de migração europeia que tradicionalmente apresentou prevalências elevadas e a transferência dessa influência através de gerações. O achado de uma prevalência mais baixa para a região Nordeste, independentemente de gênero, também não está esclarecido. O padrão regional reflete o observado nas capitais, nas quais percentuais mais elevados foram observados nas cidades da região Sul, com destaque para o sexo feminino, e em Campo Grande.

Fortes evidências científicas indicam que no mundo e no Brasil o tabagismo ativo e passivo concentra-se em populações de baixa renda que apresenta maior iniciação do que grupos populacionais com maior poder aquisitivo^5^ ^,^ ^7^ ^,^ ^11^. Condições sociais, ambientais e individuais são apontadas para explicar a maior vulnerabilidade de pessoas mais pobres. Entre essas, David et al.^4^ mencionam a menor habilidade de adolescentes em situação de desvantagem social para resistir à pressão de pais e amigos e à propaganda do tabaco, como consequência de menor competência social, autoconfiança e autoestima; maior prevalência entre pais e amigos estimulando a iniciação entre pobres; maior estresse social; maior prevalência de comorbidades psiquiátricas e de alcoolismo; e menor suporte social.

No ERICA, os únicos indicadores de condições socioeconômicas que apresentaram uma forte relação com o tabagismo foram o tipo de família e ter trabalhado no último ano. A relação com tipo de família foi observada em estudos prévios^1^ ^,^ ^11^. Sugere-se que crianças que vivem com dois pais podem ter maior estruturação emocional e maior acompanhamento por parte dos pais no sentido de reforçar a importância da abstinência nessa idade. A importância do acompanhamento dos responsáveis foi observada também na PeNSE, na qual indicadores como “pais se importariam se filho fumasse” estiveram associados à menor frequência de comportamentos de risco^6^.

A associação com trabalho pode ser explicada por dois fatores. Jovens dessa idade que trabalham, em geral, o fazem por necessidade e, portanto, apresentam baixa condição socioeconômica; adicionalmente, esses adolescentes têm renda suficiente para comprar cigarros. Essa combinação representa importante fator propulsor do consumo e mostra a importância de medidas de aumento de preços e impostos para redução do tabagismo na população em geral, sobretudo entre jovens^3^. O potencial dessas medidas foram sugeridas por Szklo et al.^14^, os quais atribuem a redução dos fumantes na faixa etária de 18 a 24 observada na PNS ao aumento de preços dos cigarros em anos recentes^a^.

Diferentemente de outros estudos realizados no Brasil, o ERICA não mostrou associação inversa entre tabagismo e escolaridade dos pais^1^ ^,^ ^6^. Uma hipótese para explicar este achado seria a de erros de classificação baseado no fato de que alguns adolescentes desconhecem qual seria o nível de escolaridade dos pais. Quase 1/5 dos adolescentes do ERICA disseram não saber ou não lembrar a escolaridade da mãe ou do pai.

Espera-se que estudar em escolas públicas seja um *proxi* de baixa condição socioeconômica e de um ambiente social que, por todas as questões relacionadas à pobreza apontadas anteriormente, poderia estar associado a fumar. Esse padrão esperado foi observado entre adolescentes do sexo feminino, mas no sexo masculino a diferença existente não foi significativa. Análises mais aprofundadas, que considerem possíveis fatores de confundimento e de modificação de efeito, poderão esclarecer melhor essas relações.

A associação com ter estado na presença de fumantes em casa e fora de casa está em consonância com a literatura^15^ ^,^ ^17^. Para os jovens, as políticas de promoção de ambientes livres do fumo justificam-se pela importância na “denormalização” e, consequentemente, menor aceitação de uma comunidade do comportamento de fumar^13^. Em 2012, o Brasil regulamentou a lei de ambientes livres do fumo que passou a ser totalmente proibida em ambientes públicos fechados^g^. O potencial dessa lei foi apontado em estudo recente no qual se observou redução significativa de exposição ao tabagismo passivo em casa e no trabalho entre os brasileiros, inclusive entre jovens^14^.

O número absoluto e a prevalência de fumantes observados neste estudo mostram que o tabagismo entre adolescentes é ainda um desafio que requer medidas de saúde pública efetivas. Visando a redução da prevalência de tabagismo entre jovens, em especial os que se encontram em situação de vulnerabilidade socioeconômica, o Brasil deve consolidar e ampliar medidas previstas na Convenção Quadro para o Controle do Tabaco de grande impacto neste subgrupo populacional. Entre essas, destaca-se a necessidade de manutenção e fortalecimento das políticas de aumento de impostos e preços e o total banimento da propaganda em pontos de venda de produtos do tabaco.

## Footnotes

a Instituto Nacional de Câncer (INCA). Observatório da Política Nacional de Controle do Tabaco. Rio de Janeiro: Instituto Nacional do Câncer; 2015 [citado 2015 set 30]. Disponível em: http://www2.inca.gov.br/wps/wcm/connect/observatorio_controle_tabaco/site/home

b Instituto Brasileiro de Geografia e Estatística. Projeção da população no Brasil por sexo e idade: 2000-2060. Rio de Janeiro; 2013 [citado 2015 set]. Disponível em: http://www.ibge.gov.br/home/estatistica/populacao/projecao_da_populacao/2013/default.shtm

c StataCorp. Stata Statistical Software: Release 14. College Station, TX: StataCorp LP; 2015.

d Panamerican Health Organization. Youth and tobacco in the Americas: results from the Global Youth Tobacco Survey 2000-2010. Washington (DC); Panamerican Health Organization [citado 2015 set 18]. Disponível em: http://www.paho.org/hq./index.php?option=com_docman&task=doc_view&gid=17483&Itemid=3482&lang=pt

e Instituto Nacional de Câncer – INCA. Vigescola. Vigilância de Tabagismo em Escolares. Dados e fatos de 17 cidades brasileiras. Instituto Nacional de Câncer, Rio de Janeiro, 2009. Disponível em: http://www.inca.gov.br/vigescola/

f Instituto Nacional de Câncer (INCA). O programa de controle do tabagismo no Brasil: avanços e desafios. Rio de Janeiro: Instituto Nacional do Câncer; 2006.

g Brasil. Decreto nº 8.262, de 31 de maio de 2014. Altera o Decreto nº 2.018, de 1º de outubro de 1996, que regulamenta a Lei nº 9.294, de 15 de julho de 1996. *Diario Oficial Uniao,* Brasília (DF), 2 junho, 2014. Seção 1, p. 108.
